# Electrical Chain Rearrangement: What Happens When Polymers in Brushes Have a Charge Gradient?

**DOI:** 10.1021/acs.langmuir.3c03127

**Published:** 2024-02-14

**Authors:** Leon A. Smook, Sissi de Beer

**Affiliations:** Department of Molecules and Materials, MESA+ Institute for Nanotechnology, University of Twente, P.O. Box 217, Enschede 7500 AE, The Netherlands

## Abstract

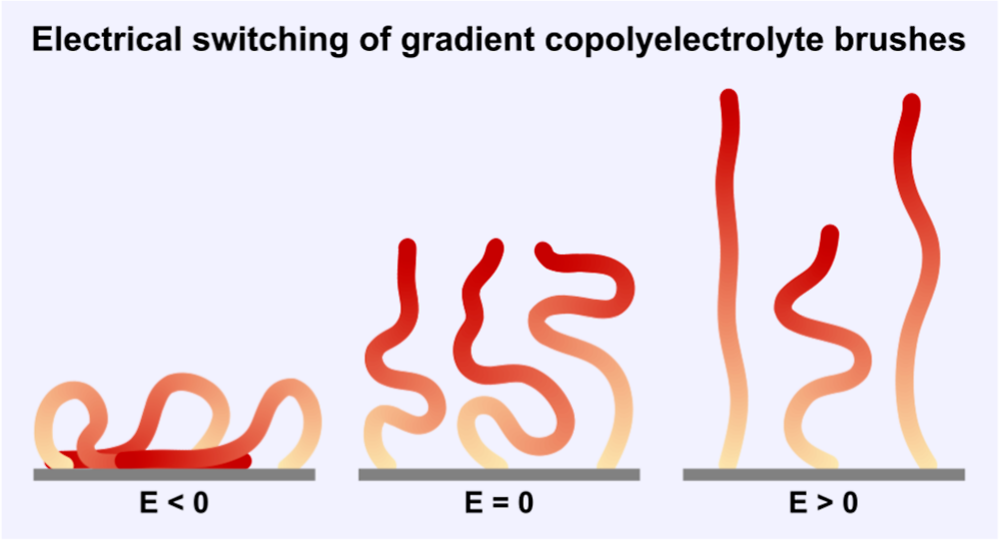

Under the influence of electric fields, the chains in polyelectrolyte brushes can stretch and collapse, which changes the structure of the brush. Copolymer brushes with charged and uncharged monomers display a similar behavior. For pure polyelectrolyte and random copolymer brushes, the field-induced structure changes only the density of the brush and not its local composition, while the latter could be affected if charges are distributed inhomogeneously along the polymer backbone. Therefore, we systematically study the switching behavior of gradient polyelectrolyte brushes in electric fields for different solvent qualities, grafting densities, and charges per chain via coarse-grained molecular dynamics simulations. Similar to random copolymers and pure polyelectrolytes, these brushes show a mixed-phase transition: intermediate states between fully stretched and collapsed are characterized by a bimodal chain-end distribution. Additionally, we find that the total charge of the brush plays a key role in the critical field required for a complete transition. Finally, we find that gradient polyelectrolyte brushes are charge-enriched at the brush–solvent interface under stretched conditions and charge-depleted under collapsed conditions, allowing for control over the local composition and thus the surface charge of the brush due to the inhomogeneous charge along the grafted chains.

## Introduction

Polyelectrolyte brushes can be used to change the functional properties of surfaces.^1−5^ The charged moieties in these brushes allow for complex interactions between the brush and its surrounding medium. Additionally, the brushes are intrinsically responsive to applied electric fields, and as a result, they can be used for many applications. For instance, these brushes can be used to control friction,^6,7^ control protein adsorption and cell adhesion,^8−14^ actuate cantilevers,^15^ tune transport properties of nanochannels,^16^ and modulate the adsorption of neutral particles.^17^

In previous work by others,^18−25^ the response of a variety of polyelectrolyte brushes to electric fields has been investigated. These brushes collapse when the charges on the chains experience an applied electric field that attracts them to the substrate, and the brushes stretch when the charges experience an applied electric field that repels them. Generally, these collapse and swelling transitions occur via a mixed-phase transition:^18,21,22,24^ A subset of the chains collapses or stretches under the influence of an electric field, while the rest of the chains approximately maintain their no-field configuration.

However, this responsiveness is limited by the charges in the coating: the higher the number of charges, the stronger the electric field has to be to achieve complete switching, so densely grafted brushes might need electric fields that are larger than the dielectric breakdown strength of the system. To work around this limitation, one can design copolymers of charged and neutral monomers in order to retain responsiveness but limit the grafted charge.

**Figure 1:**
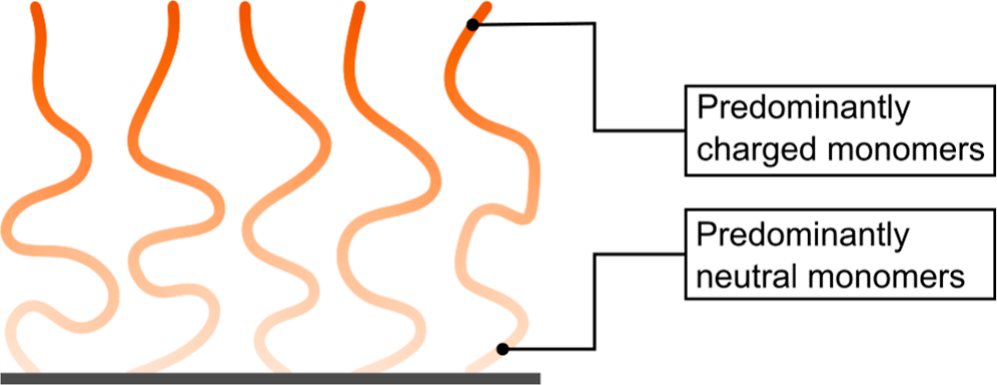
Schematic illustration of a gradient polyelectrolyte brush. Monomers near the free end are predominantly charged; monomers near the grafted end are predominantly neutral. The ratio of charged versus neutral monomers changes gradually along the polymer backbone.

We investigated the switching response of gradient polyelectrolyte brushes (see Figure 1). While a random copolymer architecture would also circumvent the limitation, we choose to investigate gradient polyelectrolyte brushes instead of other architectures. From a practical perspective, one-pot synthesis of copolymers results in copolymers with a spontaneous gradient when the monomers have different reactivities.^26−29^ Additionally, from a conceptual perspective, the gradient along the polymer backbone leads to a different local composition on one end of the polymer compared with the other. Therefore, the local composition in the system is no longer coupled only to the polymer density but also to the degree of stretching of the polymer chain. This effect provides some control over the composition of the brush near the brush–solvent interface, which could find applications in electrically mediated separations and sensing technologies. We remark that block copolymer brushes with charged and neutral blocks also decouple the charge density from the polymer density. However, these systems cannot be easily fabricated using one-pot, one-step syntheses.

Here, we investigate how polyelectrolyte brushes of chains with a charge gradient change under the influence of an external electric field. In our simulations, we systematically vary the grafting density, charge fraction, solvent quality, and electric field. We find that the electrically induced structural changes are predominantly influenced by the charge grafted to the substrate relative to the strength of the electric field. This indicates that the charge gradient in the brush only has a limited influence on the height switching of these coatings compared to random polyelectrolyte brushes or pure polyelectrolyte brushes with respect to the switching transition. Nevertheless, the gradient has an effect on the way the charges reorganize within the coating and gives some control over the local composition of the brush near the brush interface, allowing for applications where surface charge modulation is desirable.

## Model and Methods

We simulate gradient polyelectrolyte brushes with coarse-grained molecular dynamics simulations using a Kremer–Grest model.^30,31^ These brushes consist of end-anchored gradient polyelectrolyte chains from which the local composition along the polymer backbone gradually changes from predominantly neutral to predominantly charged (see Figure 1). We simulate these brushes in electric fields with different strengths in order to characterize their electric switching response.

### Description of Brushes

The simulations are performed in a simulation box with periodic boundary conditions in the lateral dimensions and fixed boundary conditions perpendicular to the grafting plane. These fixed boundary conditions are enforced by a harmonic wall potential with a spring constant of 10^3^ εσ^–2^.

We investigate the effect of three parameters on the switching behavior of gradient polyelectrolyte brushes: (1) grafting density, (2) average charge per chain, and (3) quality of the implicit solvent. For a neutral Kremer–Grest polymer in poor solvent conditions, the critical grafting density for overlap of radii of gyration of neighboring chains is ρ_crit_ = 0.06 σ^–2^ (see Section S1). For good solvent conditions and polyelectrolytes, radii of gyration are larger, so the critical grafting density is lower. Hence, we stay near the brush regime if we vary the grafting density in the range from 0.05 σ^–2^ to 0.3 σ^–2^. We vary the charge fraction per chain from 0.02 to 0.5. Finally, we vary the quality of the implicit solvent by changing the cutoff of the nonbonded interactions of both monomer types (for details see Interaction Potentials).

### Interaction Potentials

In our simulations, bonded interactions are implemented using a combination of a finite-extensible nonlinear elastic (FENE) and Weeks–Chandler–Anderson (WCA) potential
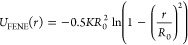
1
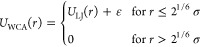
2
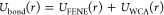
3where we set *K* = 30 εσ^–2^, *R*_0_ = 1.5 σ, ε = 1.0, and σ = 1.0. These values have shown to prevent unphysical behavior and bond crossing.^30^

The nonbonded interactions are implemented using a truncated and shifted 12–6 Lennard-Jones potential (*U*_LJ_) for neutral pair interactions and a Coulomb potential (*U*_E_) for charged pair interactions
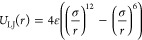
4
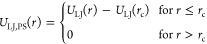
5
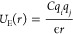
6Here, ε is the energy, σ is the zero-crossing of the Lennard-Jones potential, *q*_*i*_ and *q*_*j*_ are the charges on the interacting particles *i* and *j*, ϵ is the dielectric constant Lennard-Jones energy and dielectric constant both have epsilon as their symbolic notation. To differentiate between these quantities, we use ε for energy and ϵ for the dielectric constant. ϵ is set to 1, and *C* is an energy-conversion constant equal to 1/(4πϵ_0_). We calculate long-range charge interactions via a particle–particle/particle-mesh algorithm adjusted for 2D geometries^32,33^ with a relative accuracy of 10^–4^. The quality of the implicit solvent is adjusted via the cutoff value of the truncated Lennard-Jones potential. To model good implicit solvent conditions, we use *r*_c_ = 2^1/6^ σ which is equivalent to a repulsive WCA potential. To model poor implicit solvent conditions, we used *r*_c_ = 2.5 σ.

The simulations contain four particle types: anchors, neutral monomers, charged monomers, and counterions. All particles had a mass of 1.0. Of these particles, the charged monomers and counterions carry a charge of +1 and −1, respectively. The anchors and monomers all have the same size (σ = 1.0). Forces acting on anchoring particles are set to zero, which anchors them in place. The counterions are smaller (σ = 0.5). We model the counterions as smaller particles than the monomers because these counterions are often single atoms in experimental systems, while the monomers relate to molecular fragments of larger size. The self-and cross-interaction strengths (ε) of the nonbonded interactions is set to 1.0 for the anchors and monomers. For the counterions, the self-interaction strength is set to 0.1. The self-interaction strength is set at a low value, such that charge interactions dominate the behavior of the counterions. Cross-interaction strengths and pair-distances between the counterions and polymer are determined by using geometric mixing rules.

### Simulation Procedure

Each simulation consists of the following sequence of 5 steps. First, the initial brush structure is relaxed using energy minimization. Second, the brush is simulated in an *NVT* ensemble with a displacement limitation  using a Langevin thermostat at a reduced temperature of T* = 1.0 with a damping factor of 100 d*t* for 10^5^ time steps. In this work, the time step (d*t*) is 0.005 τ. Third, we remove the displacement limitation and continue the simulation for another 10^5^ time steps. Fourth, after initial equilibration of the brush, an electric potential is applied normal to the grafting plane so that all charged particles experience a force normal to the grafting plane
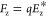
7

Under these new conditions, we continued the simulation for 2.5 × 10^5^ time steps so that the brush can equilibrate. We confirmed equilibration based on the density profiles by observing that no drift was present in the shape of these profiles. Finally, we perform a production run of 5 × 10^5^ time steps, during which we capture density profiles of the neutral monomers, charged monomers, counterions, and chain-ends. All simulations have been performed in LAMMPS.^34,35^

### Parameter Space

**Figure 2:**
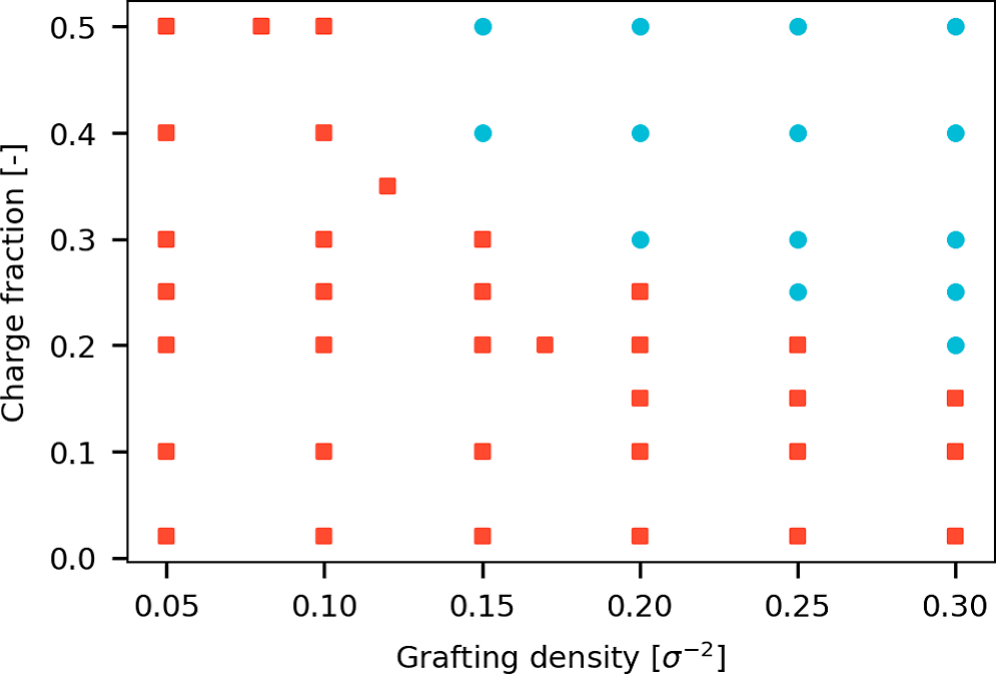
Overview of parameter combinations simulated for this work. Combinations marked with a red square (■) have been simulated under different applied electrostatic fields; combinations marked with a blue circle (●) have only been simulated without an electric field.

Our simulations do not produce stable trajectories at field strengths over 30 (|E*| > 30) for a time step of 0.005 τ. Using a mapping of ε = *k*_B_*T* = 5.7 meV and σ = 1 nm, E* = 30 is equivalent to approximately 8 × 10^8^ V/m. To prevent such unstable trajectories, we limit the field strength in our simulations to |E*| = 25. As a consequence of the limit on the field strength, we cannot achieve complete switching in the full parameter space, specifically not at high grafting densities and large charge fractions. Here, we focus our simulation efforts on systems that can achieve complete switching within the range of accessible field strengths. (For definitions of the extent of switching and critical field strength, see Section S3.) Therefore, we only simulate those systems that can achieve complete switching at multiple field strengths to save on computational resources. Those systems that have been simulated at multiple field strengths are displayed as red squares in Figure 2. The remaining systems in the parameter space have only been simulated under unperturbed conditions.

## Results and Discussion

Gradient polyelectrolyte brushes contain charged monomers and hence should respond to electric fields similar to that of pure and random polyelectrolyte brushes. However, their charge gradient may result in qualitatively and quantitatively different switching behavior. Therefore, we simulate gradient polyelectrolyte brushes using a coarse-grained model and we systematically vary grafting density, charge fraction, solvent quality, and electric field strength in the system. We studied the switching behavior of these brushes and extracted the dominant parameters to achieve complete switching. Because of numerical instability of the simulations at high field strengths, we limit our field strength to |E*| < 25. As a consequence, some systems cannot reach complete switching within the accessible field strengths; for these systems, we investigate only their unperturbed state.

### Brushes in the Absence of an Electric Field

**Figure 3:**
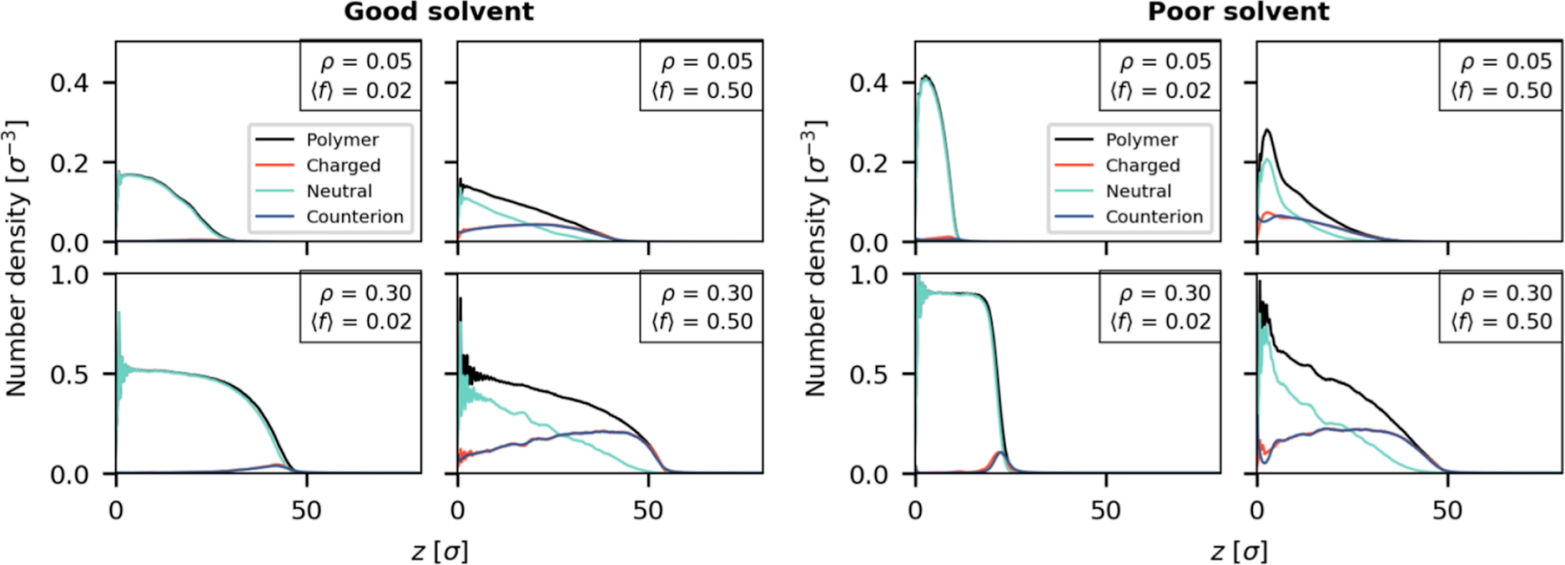
Number density profiles of four selected brushes at the extremes of the investigated parameter space. Brushes in a good solvent (left panels) and poor solvent (right panels) are higher if they contain more charge and higher grafting density. Brushes in poor solvents show a (partially) collapsed configuration. For the lowest grafted charge (ρ = 0.05 σ^–2^ and ⟨*f*⟩ = 0.02), we present rescaled density profiles for the counterion and charged monomers in Section S2.

The structure of polymer brushes is often characterized based on their density profile and height. These profiles reveal several features of the brush, such as how compact it is and whether it has a sharp interface with the solvent. The density profiles for a selection of brushes are shown in Figure 3 with good solvent conditions on the left four panels and poor solvent conditions on the right four panels. In each quadrant, we show the profiles for the brushes with the lowest and highest grafting density (ρ = 0.05 σ^–2^ and ρ = 0.30 σ^–2^) and the lowest and highest charge fraction (⟨*f*⟩ = 0.02 and ⟨*f*⟩ = 0.50). Note that the top and bottom rows have different scales for their *y*-axis.

Neutral brushes have a parabolic density profile in a good solvent, while brushes in this work have a more step-like profile in a poor solvent. The brushes in our simulations with the lowest charge (⟨*f*⟩ = 0.02) show a similar behavior. In the panels in Figure 3 corresponding to ⟨*f*⟩ = 0.02, we observe a parabolic profile in good solvent conditions and a steplike profile in poor solvent conditions. These characteristic profile shapes have been observed previously in simulations.^36−38^

The density profiles of the charged brushes show qualitatively different behavior as can be seen in the corresponding panels in Figure 3 (⟨*f*⟩ = 0.5). In all cases, the brush containing more charged monomers has a more stretched profile. Interestingly, the introduction of charged monomers leads to a linear decay in the profile for the neutral monomers higher up in the brush, and in turn this leads to a linear profile for the full polymer. Such a linearly decaying density profile is the result from charge smearing over the brush volume; this smearing effect has also been observed in pure polyelectrolyte brushes.^18,21,22,24^ Under poor solvent conditions, the change in density profile is especially profound: It goes from a mostly collapsed profile to a stretched profile with a partially collapsed segment near the grafting plane that consists predominantly of neutral monomers. In previous simulation work on polyelectrolyte brushes in poor solvent conditions,^39^ such a distinct two-layer structure was not observed, which indicates that the charge gradient may contribute to this effect. Effectively, the gradient introduces multiple regimes in the brush: One where monomers are predominantly neutral and want to aggregate due to the poor solvent conditions and one where monomers are predominantly charged and want to spread to achieve charge smearing. The introduction of charged monomers improves the effective solvent quality of the monomers, and since these charges are introduced at the free end of the chains, this leads to a pulling effect on the chains. Additionally, we note that the charged monomers reside preferentially near the brush–solvent interface as a result of their locations in the polymer chains.

### Height of Gradient Polyelectrolyte Brushes

**Figure 4:**
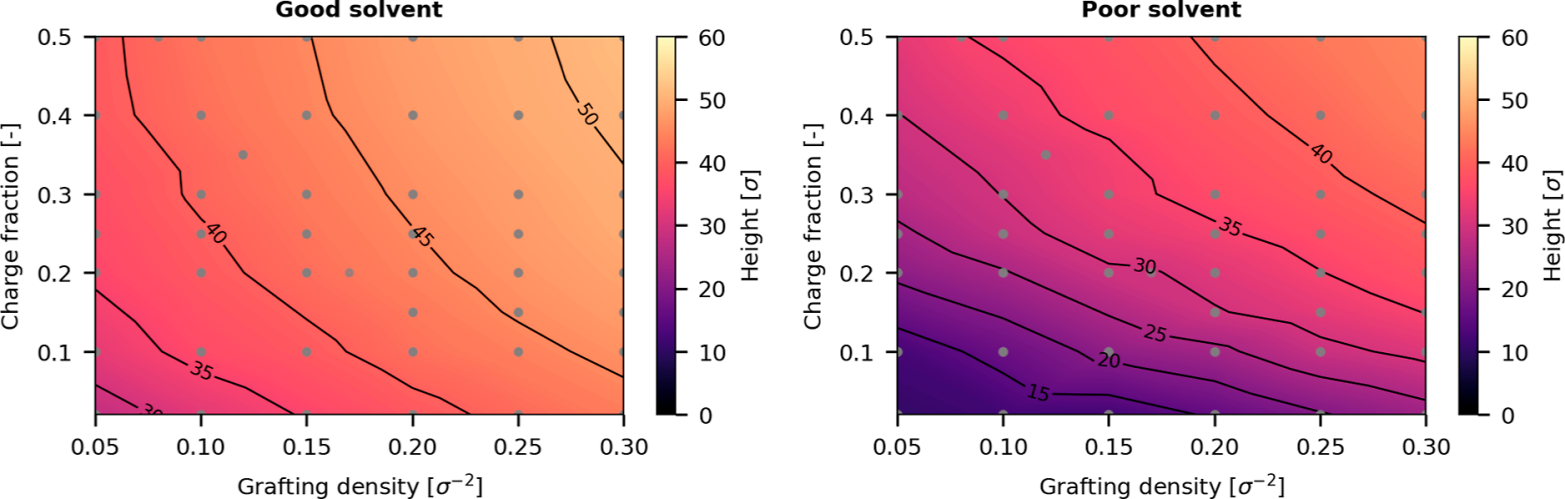
Brush height (99%-height) of gradient polyelectrolyte brush under good (left) and poor (right) solvent conditions in the absence of applied electric fields. Height scaling of these brushes agree qualitatively with the scaling behavior of polyelectrolyte brushes without a charge gradient.

Height is an important characteristic of polymer brushes, and this property is sensitive to brush properties and properties of the surrounding medium. Here, we define the height of the brush as the height below which 99% of the monomers reside ( where ϕ is the monomer density profile). The height of the brushes in unperturbed conditions is visualized in Figure 4 as a contour map where yellow indicates taller brushes and purple indicates smaller brushes. In general, brushes in good solvent conditions are taller than those in poor solvent conditions. This trend is in line with the behavior of uncharged brushes where poor solvent quality leads to a collapse of the brush. Nevertheless, in both solvent qualities, the brush height increases with increasing grafting density and increasing charge fraction.

In a salt-free environment with a good solvent, the height of a random polyelectrolyte brush depends on the interplay between several contributions to the free energy: entropy of stretching a chain, osmotic pressure of counterions, and excluded volume interactions. Not all effects contribute equally to the free energy of the system, and depending on the dominating term, different scaling regimes can be distinguished.^40^ When the osmotic pressure of the counterions dominates, the brush is in the osmotic regime and the height scales as *Naf*^1/2^. When the excluded volume interactions dominate, the brush is in the neutral brush regime and the height scales as . In these expressions, *N* is the number of segments, *a* is the monomer size, ρ is the grafting density, ν is the excluded volume parameter, and *f* is the charge fraction. We remark that the expression for the neutral brush by Csajka et al.^40^ includes the factor (1 + *f*)^2/3^ which accounts for the increase of excluded volume due to counterions in the brush. In our simulations, counterions are much smaller than monomers (ν_mon_ ≈ 8ν_ci_), so we neglect this term from our scaling and instead use an effective excluded volume parameter. The simulated brushes consist of 64 segments (*N*) with an average bond length (≈*a*) of 0.97 σ. Hence, assuming an Alexander–de Gennes brush profile, the height scales to 62.08*f*^1/2^ in the osmotic regime. Similarly, the monomers in our simulation have a Lennard-Jones diameter (*d*) of 1 σ and an excluded volume of . So, the height scales as 51.71 ρ^1/3^ in the neutral brush regime. These scaling factors are summarized in Table 1.

**Table 1:** Fitting Parameters to Model the Brush Height According to the Equation *H* = *a* + *B*ρ^1/3^ + *C*⟨*f*⟩^1/2^ + *D*ρ^1/3^⟨*f*⟩^1/2^

system type	*A*	*B*	*C*	*D*	*R*^2^
good solvent	5.74	53.16	27.47	–19.73	0.99
poor solvent	–18.06	51.45	24.65	-	0.96
osmotic brush			62.08		
neutral brush		51.71			

In Figure 4, we see that both the grafting density and charge fraction affect the brush height, so we model the brush height as a linear combination of these two regimes. Due to the connectivity between charged and neutral monomers, we expect that nonlinear effects may be needed to accurately describe the height-behavior. Therefore, we also include the interaction effect of these two scalings in the descriptive model. This model reads as follows
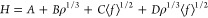
8

We fit this model to the height data (see Section S4 for details) separately for the good solvent and the poor solvent case. For the good solvent, we recover constants *A* = 5.74, *B* = 53.16, *C* = 27.47, and *D* = −19.73 (*R*^2^ = 0.99). The value for *C* is rather different from the theoretical value (62.08). This difference can be explained as follows: first, the height measure used is the 99%-height which is rather sensitive to small changes in the upper layer of the brush. Therefore, effects that make a few chains stretch have a significant effect on this value. Additionally, the model assumes block-like profiles, while the density profiles observed here are far from block-shaped. This is especially relevant for the effect of the charge fraction. An increase in charge fraction adds charge disproportionately at the already-stretched free chain end. As a result, the stretching effect will be weaker than expected since predominantly the free end will stretch and the grafted region will be less affected. This effect is reflected by the value of *C* being smaller than the value for a brush that is completely in the osmotic regime.

Additionally, in a good solvent, the interaction parameter is significant, and the prefactor has a negative value. This means that the brush height increases sublinearly if both the grafting density and charge per chain increase simultaneously. Because the gradient brushes already partially swell in a good solvent, the neutral monomer distributions are less affected by the charged profiles. As a result, there is strong coupling between the different scaling regimes. This effect is captured by the negative prefactor for the interaction.

In a poor solvent, the scaling prefactors are similar to those of the good solvent; however, the interaction term is no longer significant. The poor solvent conditions lead to a rather inhomogeneous density profile with a high density near the grafting plane and a long tail away from it. In this case, an increase in the grafting density can be seen as a polyelectrolyte chain growing from the polymer surface. Hence, a simple linear combination of these two brush regimes gives a more accurate description of the brush height.

### Response of Gradient Polyelectrolyte Brushes in Electric Fields

Electric fields exert a force on charged entities, and hence they can induce a change in polyelectrolyte brushes via the charged monomers in the brush. Here, we show how the brushes in this work respond to externally applied electric fields with different strengths. First, we describe the switching behavior of a brush in the center of our explored parameter space: ρ = 0.15 σ^–2^ and ⟨*f*⟩ = 0.20. Then, we expand our discussion with a description of the qualitative differences between brushes with higher and lower grafting densities. Finally, we develop a predictive model to describe the heights of these brushes as a function of the brush parameters.

**Figure 5:**
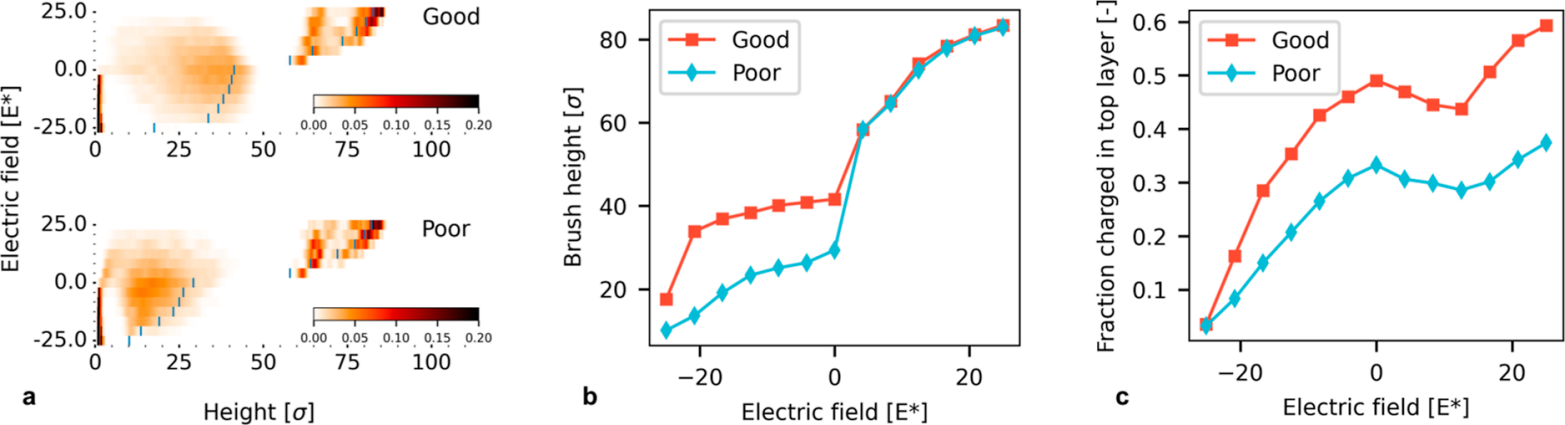
Switching characteristic of a gradient polyelectrolyte brush with ρ = 0.15 σ^–2^ and ⟨*f*⟩ = 0.20. (a) Chain end density profiles showing the end-point distribution of the chains in the brush; color scale is normalized to the grafting density (σ^–1^). (b) Brush height (99%) increases with increasing field strength with a sharp transition around E* = 0. (c) Brush composition in the top half of brush can be reduced from mostly uncharged at negative fields to overcharged with respect to ⟨*f*⟩. Snapshots of this brush are shown in Figure 6.

**Figure 6:**
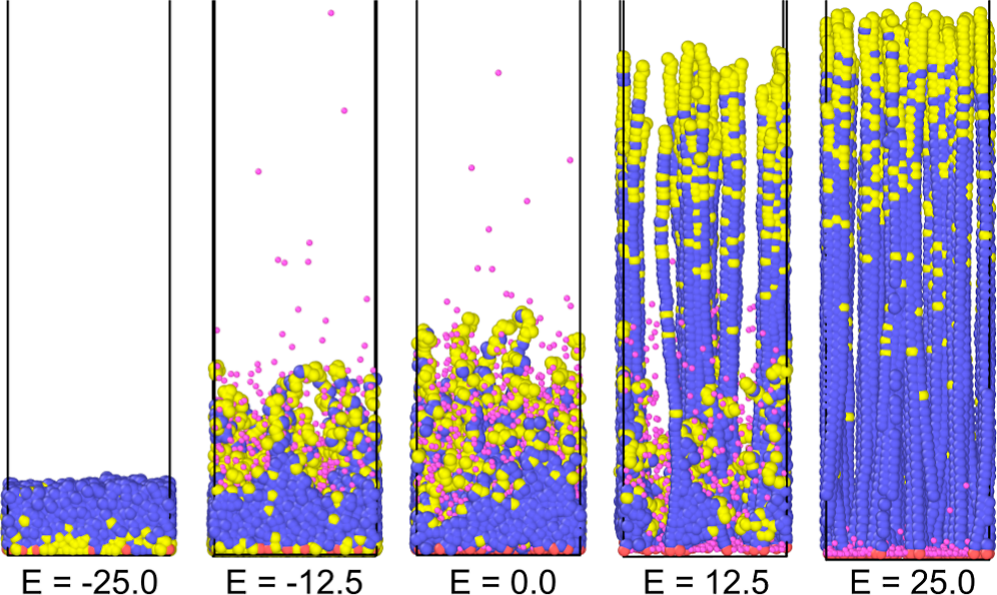
Brushes collapse and stretch via a bimodal transition. Snapshots of gradient polyelectrolyte brushes with ρ = 0.15 σ^–2^ and ⟨*f*⟩ = 0.20 for different electric field strengths. The brush parameters are the same as those in Figure 5. Colors: blue—neutral monomers, yellow—charged monomers, pink—counterions.

A gradient polyelectrolyte brush with cationic monomers collapses in a negative electric field and stretches in a positive field. In a negative electric field, the charged moieties on the chains get pulled toward the grafting plane. The chain end distribution illustrates this collapsing behavior (Figure 5a). The chain takes a more compact configuration, and the brush height decreases. With an increasing strength of the field, the chains experience a stronger attraction to the grafting plane, and more chains gain sufficient free energy to overcome excluded volume and osmotic pressure effects. Similar to pure polyelectrolyte brushes, not all chains collapse to the same degree: rather, some chains collapse fully while others remain in approximately their unperturbed configuration (Figure 6).^18,21,22,24^

The brush height does not change significantly for low-strength electric fields even though the effective grafting density of the unperturbed system reduces because the brush is in the osmotic brush regime.^41^ Only if all chains collapse does the height change drastically. Figure 5b shows how this translates to a change in the brush height. Especially for a good solvent, the brush height reduces from 33.9 σ at E* = −20.8 to nearly half of that (17.6 σ) at E* = −25.0.

When a gradient polyelectrolyte brush collapses, the composition near the brush surface changes; Figure 5c shows the composition of the top half of the brush for different strengths of the applied electric fields. The chains in the brush have their charge predominantly at their free end, which means that most charged monomers reside in the top of the brush when the chains are stretched. However, when the chains collapse due to an electric field, the charged moieties migrate toward the bottom of the brush, changing the composition of the brush surface; the fraction of charged monomers in the top half of the brush decreases from 0.49 to 0.04 in a good solvent and from 0.33 to 0.03 in a poor solvent. This internal reorganization effectively changes the surface charge and chemical nature of this surface, which could find uses in targeted separation. Hence, these gradient brushes change their chemical appearance to the surrounding medium through the application of an electric field, which allows for practical modulation of this property. A similar surface composition change under influence of an electric field has been found in diblock copolymer brushes with a charged and a neutral block.^42^

The solvent has only a marginal effect on the collapse transition (Figure 5a–c). Under poor solvent conditions and negative fields, the brush height (29.4 σ) is lower than that under good solvent conditions (41.6 σ). Excluded volume interactions dominate these systems more and the electrically induced collapse cannot reduce the brush height as much as under good-solvent conditions. This more compact configuration also leads to a slightly lower charge excess in the top of the brush under neutral conditions. Despite these differences, the qualitative behavior of switching is similar in these brushes: both the brush height and composition change gradually with increasing field strength.

If we apply a positive electric field across this coating, then the charges experience a repulsive force away from the grafting plane. As a result, the chains stretch under the influence of this field. Again, this transition has a bimodal character where only parts of the chains stretch, while others retain an approximately neutral configuration (Figure 5a). At even stronger field strengths, some chains stretch beyond their contour length, leading to the appearance of a third mode. This mode originates when fully stretched chains experience fields so strong that their bonds start to lengthen from their equilibrium value, leading to end-points at heights further from the grafting plane than the contour length of an unperturbed chain. We observe these extreme degrees of stretching in our coarse-grained model, because the bonds in the model do not have the ability to break. Stretching chains to such an extreme degree is unlikely in real-world systems, as this would lead to significant stresses on the bonds in the polymer backbone and with the substrate. It is more likely that polymers would degraft or break under such extreme conditions, since degrafting can even take place under very mild conditions depending on the grafting chemistry.^43^

The height of the brush changes drastically in a positive electric field since the stretching of just a few chains already has a significant effect on the height (Figure 5b); the height increases from 29.4 σ for a poor solvent and 41.6 σ for a good solvent under unperturbed conditions to 82.9 σ and 83.4 σ, respectively. Due to the bimodal switching character, chains stretch one-by-one with increasing field strength, and each subsequent chain contributes less to the increase of the 99% brush height. Since this behavior is sensitive to the stretching of single chains, the quality of the solvent has hardly any effect on the brush height.

The surface composition shows a minimum in the fraction of charged monomers in the top half of the brush (Figure 5c) at E* = 12.5. When only a few chains stretch, a number of neutral monomers are pulled into the top half of the brush, leading to a decrease in the fraction of charged monomers. However, at even stronger fields, when most chains are stretched, all charged monomers reside in the top half of the brush, increasing the excess charge to above the unperturbed value. In fact, the top layer charge fraction increases from 0.33 to 0.37 in a poor solvent and from 0.49 to 0.59 in a good solvent.

For all other brushes evaluated in this work, the switching transition shows characteristics similar to those displayed in Figure 6 if we consider the system going from a strong attractive field to a strong repulsive field. In a strong attractive electric field, the brush fully collapses and the ends are concentrated near the grafting plane. For slightly weaker attractive fields, only a fraction of the chains is required to neutralize the surface charges imposed by the electric field; the other chains start to assume an approximately neutral configuration. Then, in the absence of an external field, all chains assume a configuration that is similar to that encountered in other polyelectrolyte brushes. When then a slightly repulsive field is applied, a fraction of the chains stretch to counteract the electric field, while some chains remain in their neutral configuration. Finally, when a strong electric field is applied, all chains stretch.

### Critical Value for Complete Transitions

All gradient polyelectrolyte brushes show a qualitative response similar to that of an electric field. However, the magnitudes of the fields necessary to achieve this switch differ between brushes. Therefore, we fit a statistical model to the switching data in order to determine which system parameters affect the switching behavior most profoundly. First, we define the critical electric field for the swelling and the collapse transition. Then we fit linear models to this data with the grafting density (ρ) and charge fraction (⟨*f*⟩) as descriptive variables. We fit these models independently for four cases: stretching in good solvent quality; stretching in poor solvent quality; collapse in good solvent quality; and collapse in poor solvent quality.

The critical electric field is defined as the condition such that the extent of switching exceeds 95%. Specifically, we focus on the switching of the end-points since their location indicates whether a chain is stretched or collapsed. The extent of switching is defined for the stretching transition as
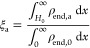
9and for the collapse transition as
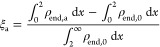
10where ρ_end_ is the end-point density, *H*_0_ the brush height under unperturbed conditions, and where the subscript *a* refers to the applied electric field. We ran simulations at discrete values for the electric field, so we fit the ξ_a_, *E*-dependence using a second-order polynomial and interpolate (or extrapolate) to ξ_a_ = 0.95 to find the corresponding critical field strength. For more details regarding this fitting procedure, we refer the reader to the Supporting Information S3.

**Figure 7:**
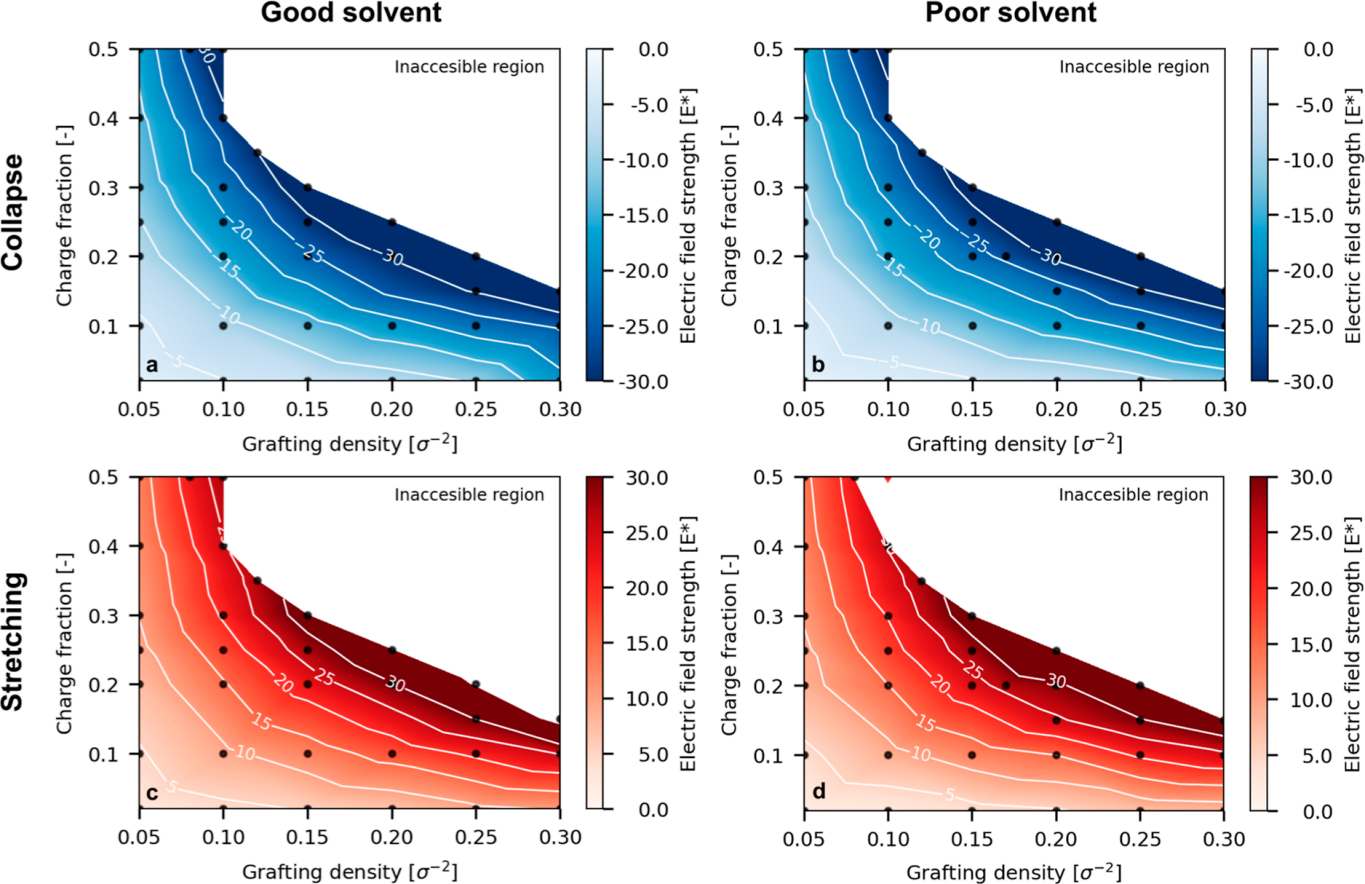
Critical electric field required to achieve a 95% extent of switching for (a) the collapse transition under good solvent; (b) the collapse transition under poor solvent conditions; (c) the stretching transition under good solvent conditions; and (d) the stretching transition under poor solvent conditions. Critical field strengths could not be reliably estimated for systems marked with a red diamond.

The switching behavior is very similar for all transitions. The critical field strength for the collapse and stretching transition are displayed as a contour plot in Figure 7 for good and poor solvent conditions. Each panel shows a transition in different conditions with the charge fraction on the *y*-axis, the grafting density on the *x*-axis, and the critical field as a color and contour line where a darker color corresponds to a stronger field. In all cases, the critical field increases with grafting density and charge fraction, specifically, the product of the two.

We fit statistical models to these critical field data to find the predominant terms that affect complete switching. We start by fitting a second order model including all terms and iteratively remove nonsignificant terms from the model. The results of this fitting procedure are summarized in Table 2. For the stretching transition, we find that the variance in the data can be well explained by the second-order model (*R*^2^ > 0.97), while for the collapse transition less of the variance can be explained (*R*^2^ < 0.96). Therefore, we fit an additional third order model to the collapse data. This extended model describes the data very well (*R*^2^ > 0.99). For a detailed description of the fitting procedure, we refer the reader to the Supporting Information S4.

**Table 2:** Prefactors of the Linear Models Fitting the Critical Field Data for the Model *E* = *a* + *B*⟨*f*⟩ + *C*ρ⟨*f*⟩ + *D*⟨*f*⟩^2^ + *E*ρ^2^ + *F*ρ^3^ + *G*ρ^2^⟨*f*⟩^a^

transition	solvent	*A*	*B*	*C*	*D*	*E*	*F*	*G*	*R*^2^
stretch	good	2.74	-	759.22	–47.41	-	-	-	0.97
stretch	poor	1.67	-	750.77	–32.81	-	-	-	0.99
collapse	good	–3.96	-	–652.18	-	-	-	-	0.93
collapse	poor	–2.51	-	–662.02	-	-	-	-	0.96
collapse	good	–2.61	17.17	–939.69	-	-	–383.53	1184.17	0.99
collapse	poor	–1.52	11.20	–790.35	-	–70.00	-	480.12	1.00

a Non-significant terms are represented with “-”. Note that all third and lower order terms that are non-significant in the descriptive models (e.g., ρ) have been excluded from the table and model equation.

In all conditions, the product of the grafting density and charge fraction contributes significantly to the value of the critical field strength even though the pure first order effects ρ and ⟨*f*⟩ are not significant. While this may appear counterintuitive, this means that effectively the critical field strength scales linearly with ρ if the prefactor includes ⟨*f*⟩, and it scales linearly with ⟨*f*⟩ if the prefactor includes ρ. The magnitude of this effect is similar for the second order models for the transition independent of the solvent condition. Hence, the total charge in the coating dominates the interaction.

For the stretching transition (Figure 7a—good solvent and Figure 7b—poor solvent), the main difference between the different solvents is found in the second-order ⟨*f*⟩^2^ term. More charge on the same chain makes it easier to reach complete switching: each additional charge on a chain means that the force pulling on the chain increases since *F* = *qE*. At the same time, the entropic penalty for stretching the chain remains the same; therefore, the same electric field would lead to a larger extension. A higher charge fraction reduces the critical electric field more in good solvent conditions than in poor solvent conditions. For a poor solvent, monomers have an energy associated with binary interactions that induce cohesion between monomers. This is not the case under good solvent conditions. This cohesion between different chains makes more energy required to pull a chain from its collapsed state. Hence, the effect of more charge per chain is weaker than that in the good solvent case.

For the collapse transition (Figure 7c—good solvent and Figure 7d—poor solvent), the quality of the second-order fit is worse than for the stretching transition, yet the grafted charge still plays a dominant role. The prefactor is slightly lower than for the stretching transition, since the chains are further away from their extension limit, so a lower force is required to move the chains in the collapsing direction. However, when we compare the predictions of this model to the observed values, we find that samples with high grafting densities are systematically underestimated and samples with high charge fraction is systematically overestimated (see Section S5). Hence, there are higher-order effects in this system that cannot be captured as a simple linear combination of ρ and ⟨*f*⟩.

We extend our model to include third-order effects and find that more of the data are described by the model (*R*^2^ > 0.99). These models include some additional interactions than just the effect of the grafted charge. Again, all models include the grafted charge as the most significant parameter. Additionally, the transition is made easier by a higher charge per chain, and a higher grafting density times grafted charge. At the same time, a higher grafting density has an adverse effect on the collapsing transition.

## Discussion

Our simulations show that we can switch gradient polyelectrolyte brushes with electric fields for a variety of grafting densities and charge per chain. This result indicates that the adsorption mediation^17^ that we found previously can be expected to happen under multiple brush and solvent conditions. As long as the interaction between the polymer and molecule is sufficiently strong to overcome thermal energy, electric fields can mediate adsorption in gradient polyelectrolyte brushes.

Similarly, this control over the brush structure can find uses in future applications for charge-based or electrically assisted separations or friction control. For instance, the charged ends of the polyelectrolyte chains can interact with proteins to form localized polyelectrolyte–protein complexes. Upon switching with an electric field, the brush can be made to collapse, which effectively forces the complex to break apart. Such a reversible processes would be similar to that shown in backbiting self-assembled monolayers.^44^

In this work, we simulated the effect of several parameters on the switching behavior of gradient polyelectrolyte brushes. Besides the parameters we explored, other parameters can be varied in our model such as the chain length, salt concentration, and monomer distribution. A full description of these effects is outside the scope of this work, yet we will provide a brief discussion of effects we expect when we vary these parameters.

For different chain lengths, we do not expect qualitative differences in the critical field strengths if we simulate different chain lengths. However, the behavior of the system during the switching transition could be significantly impacted by broad chain-length distributions. Due to the bimodal character of switching, the free energy of stretching or collapsing one chain fully is cheaper than stretching all chains partially. Since this effect is mostly entropic in nature, the entropy needed to stretch and collapse a chain of different lengths will scale with approximately the square of the chain length for small deformations. Hence, shorter chains might be more responsive to electric stimuli than longer chains, since they will lose less entropy upon deformation.

For added external salt, the salt may partially screen the electrode charge, possibly reducing the responsiveness of the brush and inducing a more complex switching behavior. The polyelectrolyte brush and the smaller, mobile salt ions could compete to screen the electrode charge. However, the grafted nature of the polyelectrolyte may result in complex behavior in these systems. To understand how these polyelectrolyte brushes switch in practical settings, it will be paramount to study their switching behavior in electrolyte solutions to see what effect free ions have on chain and brush rearrangements.

## Conclusions

We showed that gradient polyelectrolyte brushes switch under the influence of an external electric field: they stretch when the field repels the charged monomers and they collapse when the field attracts them. Here, we performed coarse-grained molecular dynamics simulations on a Kremer–Grest polymer with charged monomers. In these simulations, we recovered a switching behavior similar to polyelectrolyte brushes without a gradient in the charge along the chain: for intermediate electric field strengths, we observed partitioning of the brush into multiple populations of collapsed/stretched chains and relaxed chains. Additionally, a statistical fit of the data showed that the grafted charge in the brush is the dominating factor that influences the field strength needed for the complete collapse and swelling. These descriptions allow us to design these coatings with switchability in mind both in future experimental work and in future simulations.

Additionally, the surface composition of the gradient polyelectrolyte brush can be switched, while such a change is not possible with polyelectrolyte brushes without a charge gradient. Under stretched conditions, the brush surface is overcharged compared to the average charge fraction, while under collapsed conditions, the brush surface is undercharged and in some cases even tends to a neutral surface. Hence, when polymers in brushes have a charge gradient, their chain rearrangement in electric fields influences how these coatings express themselves toward the medium.

## Supporting Information Available

Estimate of critical grafting density in poor solvent; enlarged density profiles for low grafted charge; determination of critical electric field strength; fitting descriptive models to simulation data; and fit quality of second order model on collapse transition (PDF)
