# Supplementary material for: Electrical Chain Rearrangement: What Happens When Polymers in Brushes Have a Charge Gradient?
Source: Langmuir. 2024 Feb 14;40(8):4142–51. doi: 10.1021/acs.langmuir.3c03127 (PMC10906002; doi:10.1021/acs.langmuir.3c03127)
Supplement: Supplementary file 1 — la3c03127_si_001.pdf [file la3c03127_si_001.pdf]

Supplementary Information: Electrical Chain Rearrangement: What Happens When Polymers in Brushes Have a Charge Gradient?

Leon A. Smook* and Sissi de Beer

December 2023

Department of Molecules & Materials
MESA+ Institute
University of Twente
P.O. Box 217
7500 AE Enschede
The Netherlands

Contents

S1 Estimate of critical grafting density in poor solvent S2

S2 Enlarged density profiles for low grafted charge S3

S3 Determination of critical electric field strength S4

S4 Fitting descriptive models to simulation data S5

S5 Fit quality of second order model on collapse transition S6

*Email: l.a.smook@utwente.nl

S1 Estimate of critical grafting density in poor solvent

In order to estimate the critical grafting density in our system under poor solvent conditions, we assume that the Kremer-Grest chain takes up a space-filling configuration. The critical grafting density can then be found by comparing the radius of the polymer globule to the interchain distance.

The volume associated with a chain can be estimated from the volume of the monomers and counterions:

$$V = N\frac{4}{3}\pi\left(\left(\frac{d_{\mathrm{m}}}{2}\right)^3 + f\left(\frac{d_{\mathrm{ci}}}{2}\right)^3\right) \tag{S1}$$

This gives a radius of a space filling sphere that reads:

$$r = \left(\frac{3V}{4\pi}\right)^{\frac{1}{3}} = \left(N\left(\left(\frac{d_{\mathrm{m}}}{2}\right)^3 + f\left(\frac{d_{\mathrm{ci}}}{2}\right)^3\right)\right)^{\frac{1}{3}} \tag{S2}$$

In our simulations, we have the following parameters: $N = 64$, $d_{\mathrm{m}} = 1.0\ \sigma$ and $d_{\mathrm{ci}} = 0.5\ \sigma$, and $f \in [0.02, 0.50]$. This gives that the radius of the polymer can be expressed as:

$$r = 2.00\ \sigma \quad \text{for} \quad f = 0.02 \tag{S3}$$

$$r = 2.04\ \sigma \quad \text{for} \quad f = 0.50 \tag{S4}$$

Next, we determine the critical grafting density (ρ_{crit}) using:

$$\rho_{\mathrm{crit}} = \frac{1}{(2r)^2} \tag{S5}$$

and we find a critical grafting density of $\rho_{\mathrm{crit}} \in [0.0600, 0.0626]\ \sigma^{-2}$ for the brushes in this work.

S2 Enlarged density profiles for low grafted charge

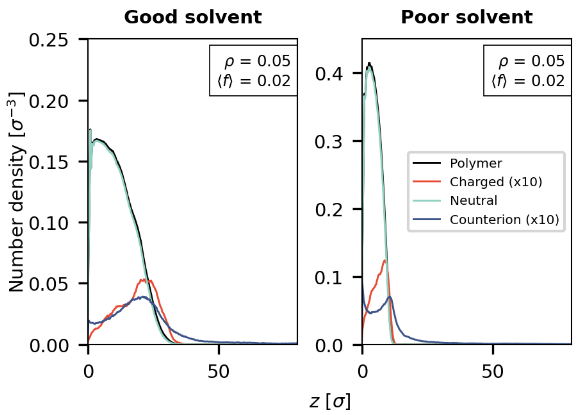

Figure S1: Number density profiles of $\rho = 0.05\ \sigma^{-2}$ and $\langle f \rangle = 0.02$ in good and poor solvent where ionic moieties are multiplied by a factor 10 for visibility.

S3 Determination of critical electric field strength

In order to determine the critical electric field, we first determine the extent of switching of the coating for the electric field it has been exposed to.

Stretching We define the extent of switching as the number of chain ends that extent above the brush height of a brush in the absence of an applied field, which can be captured in the following expression:

$$\xi_a = \frac{\int_{H_0}^{\infty} \rho_{\mathrm{end,a}} \mathrm{d}x}{\int_{0}^{\infty} \rho_{\mathrm{end,0}} \mathrm{d}x} \tag{S6}$$

where H_0 is the height below which 99% of the monomers are present when **no** electric field is applied, and ρ_{end} is the end-point number density profile of the brush. Based on this expression, we can calculate the extent of switching for all coatings exposed to a stretching electric field.

Collapsing We can again define an extent of switching. Where for the stretching transition we can simply define stretching as chain ends moving away from the neutral configuration, the definition for the collapsing transition is a little more involved. Here we define this transition as:

$$\xi_a = \frac{\int_{0}^{2} \rho_{\mathrm{end,a}} \mathrm{d}x - \int_{0}^{2} \rho_{\mathrm{end,0}} \mathrm{d}x}{\int_{2}^{\infty} \rho_{\mathrm{end,0}} \mathrm{d}x} \tag{S7}$$

where all definitions are equal to those in Equation S6. We define a collapsed chain as a chain with an end-point lower than $z = 2\ \sigma$. Based on this definition, we can again predict the critical field of switching under both solvent conditions.

For each combination of parameters, these two expressions provide us with a data set of ξ_a versus E^*. To this data, we fit a second order model using ordinary linear regression. We extrapolate or interpolate to $\xi_a = 0.95$ and find the corresponding electric field strength. This field strength is determined to be the critical electric field strength for the transition.

S4 Fitting descriptive models to simulation data

In order to fit a linear model to the data, we curate a data set from the simulation output that includes:

1. the response parameter (height H, or critical field strength E^*_{crit})
2. the predictor variables (grafting density ρ, charge fraction f)

If desired, we extend the predictor variables with linear combinations so that cross-interactions and higher order terms are included (e.g. ρ^2 or ρf^2). For these higher order model, we reduce the model to only significant terms with the following procedure:

1. Fit the higher-order model to the data;
2. Evaluate the significance of each term in the model;
3. Find the term with the lowest significance;
    - If $p > 0.05$ for the least significant term: exclude this term from the model and the repeat steps 1. through 3.
    - If $p < 0.05$ for the least significant term: keep the current model and report the fit results.

Height model We fit the height model based on scaling laws from literature [1] that include the relationships $H \propto \rho^{1/3}$ and $H \propto f^{1/2}$. We fit a linear model to the data with the height as the response variable and $\rho^{1/3}$ and $f^{1/2}$ as the predictor variables via ordinary least squares.

Critical field To model the critical electric field strength, we use the evaluated critical field strength E^*_{crit} as the response variable and ρ and f as the predictor variables. First, we fit a second-degree model to the data. If this model does not provide a good fit to the data, we extend the model with third-degree terms. In both cases, we eliminate non-significant terms using the previously mentioned procedure.

S5 Fit quality of second order model on collapse transition

The second-order model does not describe the data of the collapse transition well. The R^2-value of this fit is a first indication as they are only 0.96 (poor solvent) and 0.93 (good solvent). Additionally, when plotting the predicted values versus the observed values one can see a decent correlation between both (as indicated by the R^2). However, we see a systematic error in the model if we visualize the different brush properties with different makers and link the marker size to the charge fraction and the marker transparency to the grafting density as shown in Figure S2 for the good solvent and Figure S3 for the poor solvent. More transparent and larger markers appear below the line of perfect predictions (dotted diagonal), while smaller, more opaque markers appear above this line. Hence, the model contains a systematic misprediction and higher order terms might be a valuable addition to remove this systematic error.

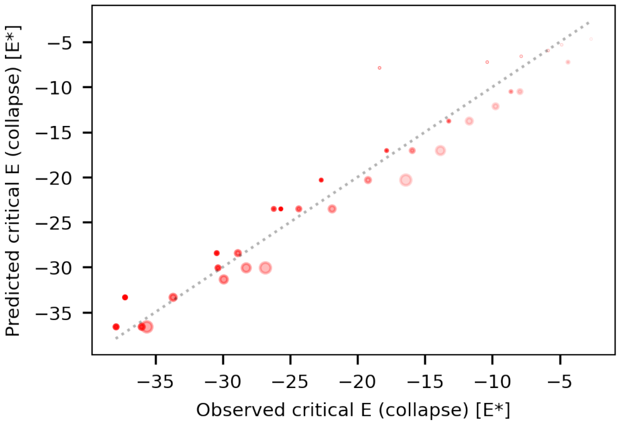

Figure S2: Predicted versus observed values for the second order model fit to the critical field data for good solvent conditions. Larger markers correspond to higher charge fractions and more transparent markers to higher grafting densities.

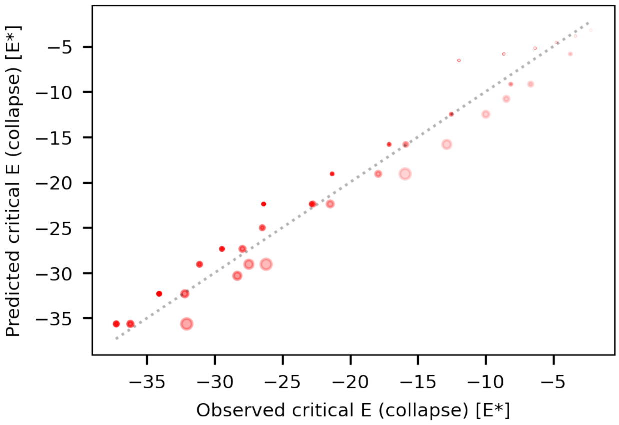

Figure S3: Predicted versus observed values for the second order model fit to the critical field data for poor solvent conditions. Larger markers correspond to higher charge fractions and more transparent markers to higher grafting densities.

References

1. Csajka, F., Netz, R., Seidel, C. & Joanny, J.-F. Collapse of Polyelectrolyte Brushes: Scaling Theory and Simulations. *The European Physical Journal E* **4,** 505–513 (2001).
